# Prognostic value of the long noncoding RNA *HOTTIP* in human cancers

**DOI:** 10.18632/oncotarget.19166

**Published:** 2017-07-11

**Authors:** Wei Li, Na Li, Xinmei Kang, Ke Shi, Qiong Chen

**Affiliations:** ^1^ Department of Geriatrics, Xiangya Hospital of Central South University, Changsha, Hunan Province, People's Republic of China; ^2^ Department of Pathology, The First Affiliated Hospital of Hunan University of Medicine, Huaihua, Hunan Province, People's Republic of China

**Keywords:** HOTTIP, lncRNA, cancer, clinical outcome, meta-analysis

## Abstract

Human Homeobox A transcript at the distal tip (HOTTIP) is a putative oncogene in solid tumors. We performed a meta-analysis to investigate the association between HOTTIP expression and clinical outcomes in cancer patients. Eligible studies were collected from a literature search of the online electronic databases of Embase, Web of Science, PubMed and the China National Knowledge Infrastructure (up to January 2, 2017). Fixed-effects models were used to compute pooled odds ratios (ORs) and hazard ratios (HRs). In total, we analyzed nine studies that included 800 patients with seven tumor types. Overall survival was lower for patients with high HOTTIP expression than for those with low expression (HR = 2.30, 95% confidence interval [CI]: 1.81–2.91, *P* < 0.001). High HOTTIP expression was also associated with lymph node metastasis (OR = 2.40, 95% CI: 1.70–3.37, *P* < 0.001), distant metastasis (OR = 3.30, 95% CI: 1.78–6.12, *P* < 0.001), poor tumor differentiation (OR = 1.55, 95% CI: 1.03–2.32, *P* = 0.036) and a poor clinical stage (OR = 3.28, 95% CI: 2.22–4.83, *P* < 0.001). This meta-analysis demonstrated that high HOTTIP expression in cancer patients is associated with poor clinical outcomes. Thus, HOTTIP is a potential predictive biomarker of cancer.

## INTRODUCTION

Cancer is a major health problem all over the world, as the second leading and leading cause of death in the US and China, respectively [1, 2]. Although diagnostic and treatment modalities have been developed for decades, the average five-year survival rate for cancer patients is still very low. The main reasons for the high cancer mortality rate are the lack of effective detection methods at early stages and the high recurrence rate. Therefore, the study of diagnostic and prognostic markers in cancer patients has major clinical significance.

Long noncoding RNAs (lncRNAs) are RNA molecules ranging from 200 nucleotides to 100 kilobases in length, without protein-coding abilities [3, 4]. LncRNAs are deregulated in a variety of diseases, including cancer [5–8]. These differentially expressed RNAs participate in various regular biological processes at the transcriptional and posttranscriptional levels [5]. LncRNAs can regulate gene expression through multiple approaches, such as by recruiting transcription factors or directly binding to proteins, RNA and DNA [5]. Many lncRNAs have been reported to participate in tumorigenesis, tumor progression and cancer prognosis [9–12].

Homeobox A transcript at the distal tip (*HOTTIP*) is a 3,764-nucleotide-long lncRNA encoded by a gene at the 5’ end of the *HOXA* cluster (chromosome 7p15.2) [4]. *HOTTIP* is upregulated in various carcinomas, such as lung carcinoma [13], gastric carcinoma (GC) [9], pancreatic carcinoma [11], hepatocellular carcinoma (HCC) [8] and squamous cell carcinoma [14]. In addition, *HOTTIP* levels have been associated with tumor pathological characteristics and patient prognostic outcomes [10–14]. These findings suggest that *HOTTIP* could be developed as a potential diagnostic and prognostic biomarker for human cancers. However, individual studies may be inaccurate or inadequate due to limitations of the sample sizes or research programs. Therefore, in this study, we collected all relevant publications and conducted a meta-analysis to explore the relationship between *HOTTIP* expression and cancer clinical outcomes, and to determine the potential of *HOTTIP* as a common predictive biomarker for metastasis and prognosis in cancer patients.

## RESULTS

### Study characteristics

**Figure 1:**
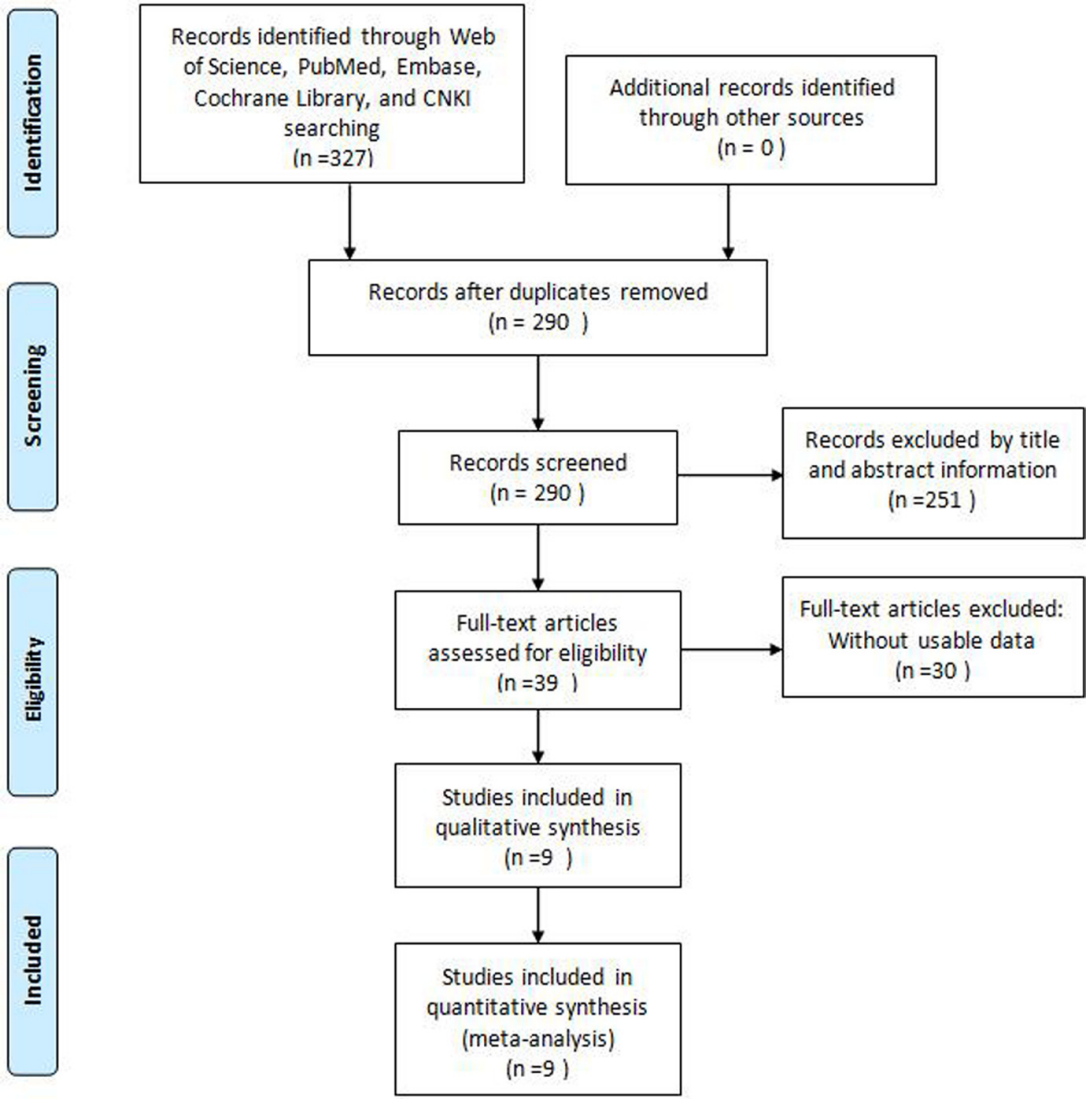
Flow diagram of the literature search and selection

Our initial search retrieved 327 potentially relevant papers from Embase, Web of Science, PubMed and the China National Knowledge Infrastructure (CNKI). Following the removal of duplicates, 290 records were preserved. After the titles and abstracts were screened, 39 full-text articles remained. However, 30 of them were excluded due to incomplete data. Ultimately, nine studies met the eligibility criteria and were included in the meta-analysis [8–12, 13, 15–17]. A flow diagram of the study selection process is shown in Figure 1.

In total, 800 patients were included in this study. Eight studies came from China and one came from Switzerland. Cancers of seven carcinoma types were included in our study: HCC, osteosarcoma (OSA), colorectal cancer (CRC), pancreatic cancer (PC), GC, tongue squamous cell carcinoma (TSCC) and breast cancer (BC). In all of the studies, lncRNA *HOTTIP* expression was detected by quantitative reverse transcription PCR (qRT-PCR). The main features of the nine studies that qualified for the present meta-analysis are summarized in Table 1.

**Table 1:** Main characteristic of the eligible studies

Study	Region	Tumor type	Sample size	Test method	Cut-off	*HOTTIP* expression						Outcome measure	Follow-up (months)
						High with LNM	High with DM	High	Low with LNM	Low with DM	Low		
Quagliata 2014	Switzerland	HCC	52	qRT-PCR	ROC curve	NA	NA	32	NA	NA	20	OS	Over 80
Ge 2015	China	HCC	48	qRT-PCR	ROC curve	NA	NA	NA	NA	NA	NA	OS	18 (5–72)
Li 2015	China	OSA	68	qRT-PCR	Median value	NA	11	34	NA	3	34	OS	Over 60
Ren 2015	China	CRC	156	qRT-PCR	Median value	47	27	77	36	14	79	OS	46 (33–65)
Wang 2015	China	PC	144	qRT-PCR	ROC curve	75	NA	118	10	NA	26	OS	Over 60
Zhang 2015	China	TSCC	86	qRT-PCR	Median value	28	7	44	18	1	42	OS	38 (23–60)
Lian 2016	China	CRC	48	qRT-PCR	X-tile algorithm	21	NA	32	7	NA	16	NA	NA
Ye 2016	China	GC	98	qRT-PCR	Median value	39	NA	49	29	NA	49	OS	Over 60
Yang 2017	China	BC	100	qRT-PCR	Median value	32	NA	50	19	NA	50	OS	Over 100

ROC, receiver operating characteristic. NA, not applicable.

### Relationship between increased *HOTTIP* expression and overall survival

**Figure 2:**
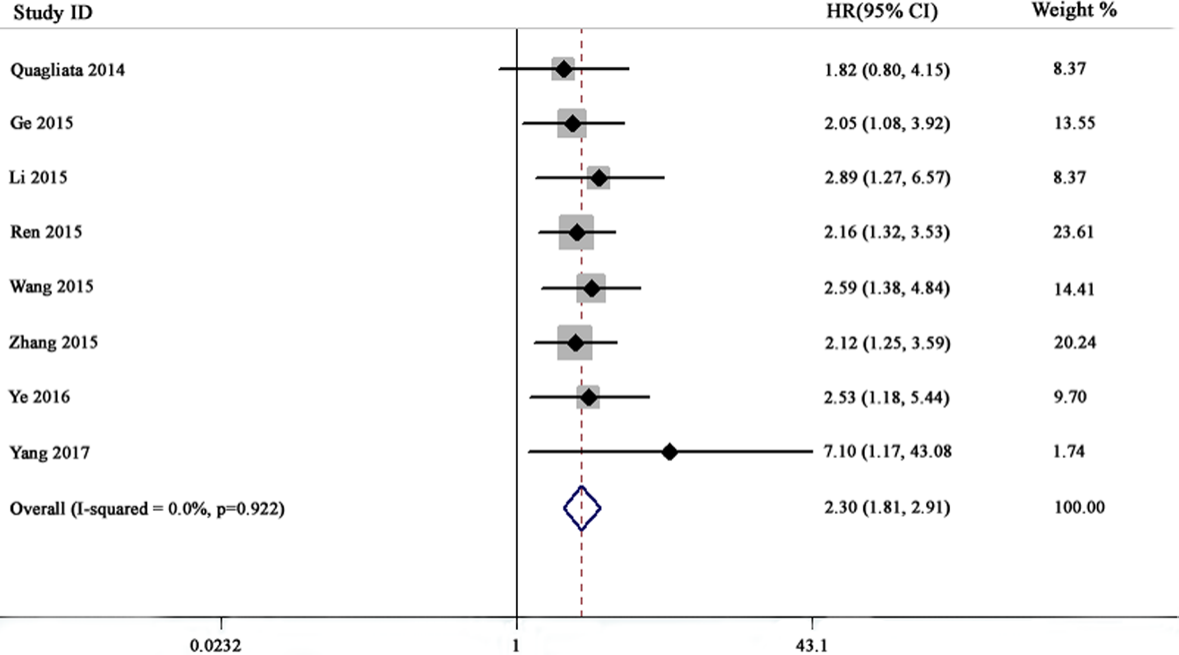
Forest plot for the relationship between *HOTTIP* expression and OS

**Figure 3:**
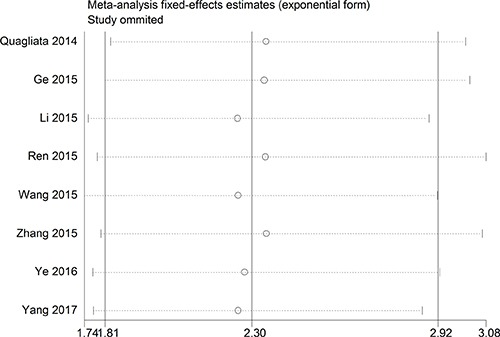
Sensitivity analysis for the meta-analysis

The relationship between *HOTTIP* expression and overall survival (OS) was evaluated in eight studies including 752 patients. No significant heterogeneity was observed among the studies (*I*^2^ = 0%, *P* = 0.922), so a fixed-effects model was used to pool the results. The pooled HR indicated that *HOTTIP* expression was negatively associated with OS (HR = 2.30, 95% confidence interval [CI]: 1.81–2.91, *P* < 0.001, fixed-effects model) (Figure 2). To evaluate the robustness of the merged results, we conducted a sensitivity analysis by successively omitting each individual study. The results were not significantly affected by the exclusion of any individual study, indicating that the results of this meta-analysis were robust (Figure 3).

### Relationship between increased *HOTTIP* expression and clinicopathological parameters

**Figure 4:**
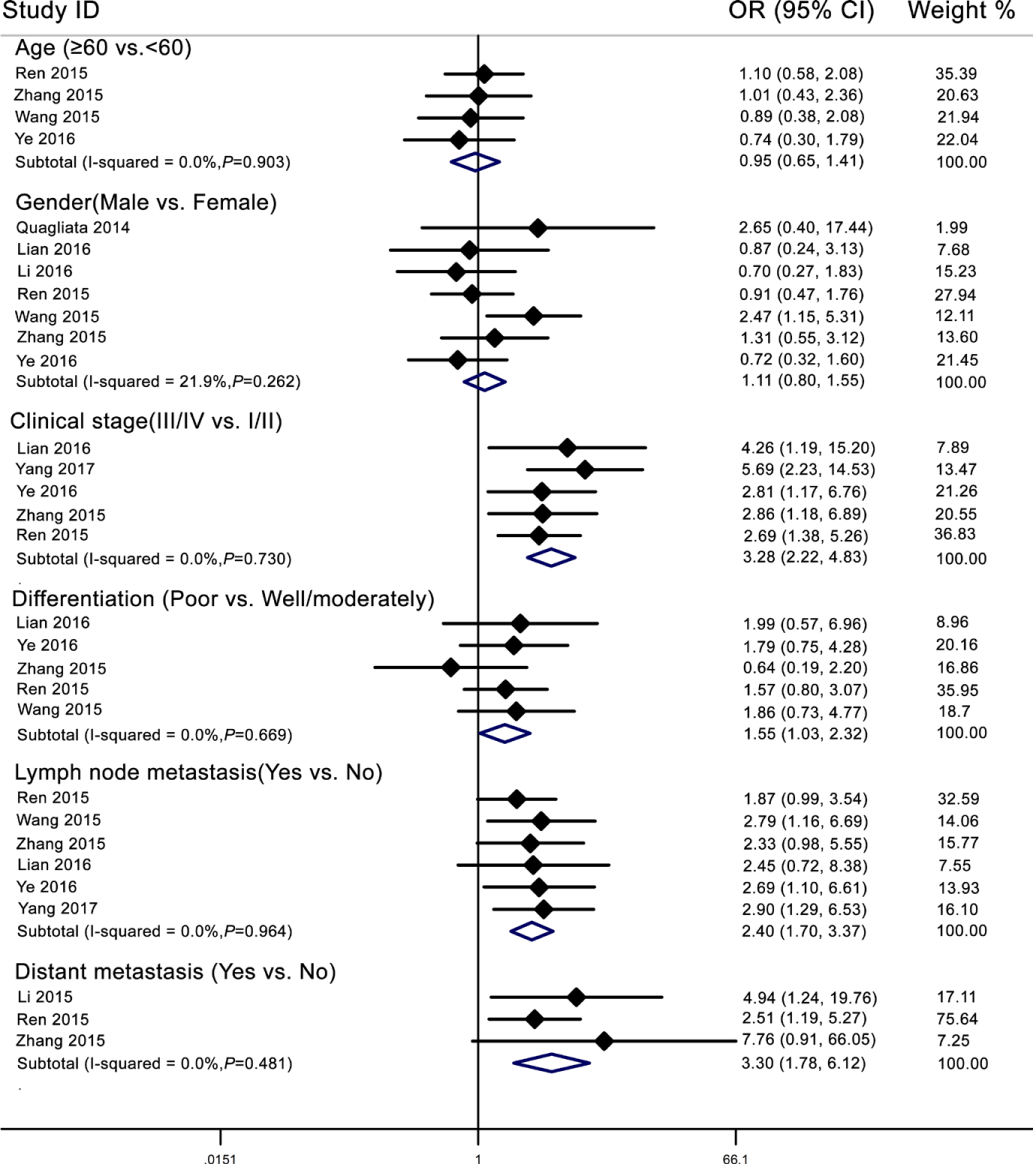
Forest plot for the relationship between *HOTTIP* expression and clinicopathological parameters

Six articles encompassing 632 patients evaluated lymph node metastasis (LNM) according to lncRNA *HOTTIP* expression. There was no significant heterogeneity among these studies (*I*^2^ = 0%, *P* = 0.964). Subsequently, a fixed-effects model was adopted to analyze the data (Figure 4, Table 2). The odds ratio (OR) of LNM for the high *HOTTIP* expression group versus the low expression group was 2.40 (95% CI: 1.70–3.37, *P* < 0.001).

**Table 2:** Meta-analysis results on the association of increased *HOTTIP* expression with clinicopathological parameters

Clinicopathological parameter	Patient number	OR (95% CI)	*P* value	Heterogeneity		
				*Q*	*I*^2^	*P*_h_
Age (≥ 60 vs.< 60)	484	0.95 (0.65–1.41)	0.81	0.57	0.0%	0.902
Gender (Male vs. Female)	662	1.11 (0.80–1.55)	0.536	7.69	21.9%	0.261
Clinical stage (III/IV vs. I/II)	488	3.28 (2.22–4.83)	< 0.001	2.03	0.0%	0.731
Differentiation (Poor vs. Well/moderately)	532	1.55 (1.03–2.32)	0.036	2.37	0.0%	0.667
Lymph node metastasis (Yes vs. No)	632	2.40 (1.70–3.37)	< 0.001	0.98	0.0%	0.964
Distant metastasis (Yes vs. No)	310	3.30 (1.78–6.12)	< 0.001	1.46	0.0%	0.481

Three studies described cancer patients with distant metastasis (DM). There was no heterogeneity among these studies (*I*^2^ = 0%, *P* = 0.481). Thus, a fixed-effects model was used to calculate the pooled result (Figure 4, Table 2). The OR of DM was 3.30 for patients with high *HOTTIP* expression (95% CI: 1.78–6.12, *P* < 0.001). These aggregated results suggested that cancer patients with high *HOTTIP* expression were more susceptible to LNM and DM than patients with low *HOTTIP* expression.

In addition, we found that high *HOTTIP* expression was positively associated with poor tumor differentiation (OR = 1.55, 95% CI: 1.03–2.32, *P* = 0.036) and a poor clinical stage (OR = 3.28, 95% CI: 2.22–4.83, *P* < 0.001) (Figure 4, Table 2). However, increased *HOTTIP* expression did not correlate significantly with age or gender (Figure 4, Table 2). Due to insufficient data, we were unable to determine the association between high *HOTTIP* expression and other clinicopathological parameters.

### Publication bias

**Figure 5:**
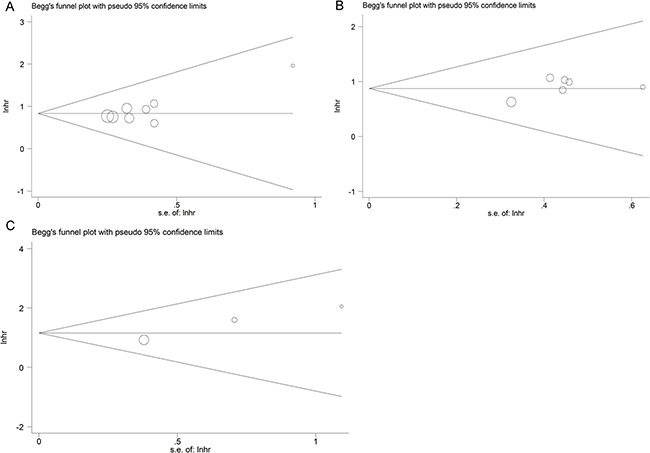
Funnel plot analysis of potential publication bias in the meta-analysis (**A**) OS group; (**B**) LNM group; (**C**) DM group.

We performed Begg's funnel plot analysis to evaluate the publication bias. There was no obvious publication bias affecting the analysis of OS (*Pr*>|z|=0.458), LNM (*Pr*>|z|=0.851) or DM (*Pr*>|z|=0.296) (Figure 5).

## DISCUSSION

*HOTTIP* is a 3,764-nucleotide-long lncRNA encoded by a gene at the homeobox A (*HOXA*) locus [4]. *HOTTIP* recruits histone-modifying enzymes to activate *HOX* gene transcription and inhibit tumor-suppressor gene expression [4, 18]. Therefore, *HOTTIP* has been considered as a carcinogenic factor, and overexpression of *HOTTIP* has been reported to promote tumor growth [13, 18]. We conducted this comprehensive meta-analysis to evaluate the relationship of lncRNA *HOTTIP* expression with prognostic results and clinicopathological parameters in cancer patients.

Nine eligible studies met the selection requirements of this meta-analysis. The data from tumor patients were processed according to OS, age, gender, clinical stage, differentiation, LNM and DM. Our results suggested that cancer patients with high *HOTTIP* expression in tumor tissues were more likely to exhibit poor tumor differentiation, a poor clinical stage, LNM and DM than patients with low *HOTTIP* expression. In addition, we found that increased *HOTTIP* levels were significantly and negatively associated with shorter OS times in tumor patients. Sensitivity analysis indicated that no single study impacted the overall results, suggesting that the pooled results were robust. Furthermore, no obvious publication bias or heterogeneity was observed among our included studies.

Many studies have demonstrated that *HOTTIP* may be an oncogenic lncRNA. *HOTTIP* was reported to influence HCC cell proliferation [8]. Knockdown of *HOTTIP* reduced *HOXA13* expression, and knockdown of *HOXA13* also reduced *HOTTIP* expression in HCC-derived cell lines [8]. Tsang and colleagues [19] found that downregulation of *HOTTIP* markedly reduced the expression of numerous *HOXA* genes and weakened liver cancer growth and metastasis both *in vivo* and *in vitro*. *HOTTIP* also was shown to be upregulated in human pancreatic ductal adenocarcinoma tissues and to promote pancreatic ductal adenocarcinoma cell proliferation, migration, invasion and resistance by regulating *HOXA13* [20]. However, another study in pancreatic cancer cells demonstrated that *HOTTIP* did not modulate *HOXA13* expression, but did regulate the expression of other *HOX* genes such as *HOXA1*, *HOXA9*, *HOXA10*, *HOXA11* and *HOXB2* [21]. In addition, Lian and coworkers [12] found that *HOTTIP* promoted CRC cell growth by silencing p21 expression. Furthermore, *HOTTIP* was reported to inhibit cell apoptosis and promote the progression of prostate cancer and lung cancer [13, 22]. These results may illustrate why high *HOTTIP* expression was significantly associated with a poor prognosis in cancer patients in this meta-analysis.

There are some limitations that should be noted for the current study. Firstly, most of the included cancer patients were from Chinese populations. Thus, our results need to be confirmed with more clinical data from other ethnic groups. Secondly, some HRs and their corresponding 95% CIs were extracted from Kaplan-Meier curves, and may have been less reliable than those obtained directly from articles. Thirdly, the number of original studies included in this meta-analysis was small. Fourthly, studies with positive results are more likely to be published than those with negative results. Thus, as a literature-based analysis, this study may have overestimated the association between high *HOTTIP* expression and clinical outcomes. Finally, many other factors may also influence OS, such as gender, age and treatment. Thus, the results of our meta-analysis should be confirmed in future studies.

In conclusion, our meta-analysis suggests that the upregulation of *HOTTIP* is a risk factor for poor clinical outcomes in diverse cancers, and could serve as a potential prognostic biomarker. Nevertheless, given the limitations described above, well-designed studies of particular carcinoma types and large sample sizes are needed to confirm the clinical utility of *HOTTIP*.

## MATERIALS AND METHODS

### Literature search strategy

We conducted a literature search in the online electronic databases of Embase, Web of Science, PubMed and CNKI (up to January 2, 2017). The search keywords and their combinations were: “HOTTIP OR Homeobox A transcript at the distal tip” AND “cancer OR carcinoma OR tumor OR tumour OR neoplasm”. Only English and Chinese articles were included in this study.

### Selection and exclusion criteria

The inclusion criteria of this meta-analysis were: (1) research on the association between *HOTTIP* and cancer prognosis, (2) division of patients into two groups: high or low *HOTTIP* expression, and (3) sufficient data for the computation of ORs or HRs with 95% CIs. The exclusion criteria for our meta-analysis were: (1) duplicate articles, (2) case reports, letters, expert opinions, editorials and reviews, and (3) studies without available data.

### Data extraction

Two investigators (Wei Li and Xinmei Kang) extracted and reviewed the data from the eligible studies independently. Another researcher (Ke Shi) resolved any disagreements. The collected data were as follows: first author's name, publication date, study region, tumor type, tumor stage, detection method for *HOTTIP* expression, criteria for high *HOTTIP* expression, sample size, total patient number, number of patients in the high *HOTTIP* and low *HOTTIP* groups, number of patients with LNM and DM in each group, survival analysis, follow-up period, HR and corresponding 95% CI. If the survival data were not shown directly in the article, Engauge Digitizer v.4.1 software was used to obtain them from the Kaplan-Meier curve, as previously described [23].

### Statistical methods

STATA 12.0 software (Stata, College Station, Texas) was used to perform all statistical analyses in this study. Patients were separated into the high and low *HOTTIP* expression groups according to the original published articles. The heterogeneity among the included studies was judged with the *Q*-statistic test and the chi-squared test. A “Begg's funnel plot” was used to determine the potential publication bias. A fixed-effects model was used to analyze the pooled results when the included studies did not exhibit significant heterogeneity (*P* > 0.1); otherwise, a random-effects model was employed (*P* < 0.1). A sensitivity analysis was conducted to evaluate the robustness of the overall results. All *P*-values were determined with a two-tailed test, and *P* < 0.05 was regarded as statistically significant.

## References

[1] Siegel RL, Miller KD, Jemal A (2016). Cancer statistics, 2016. CA Cancer J Clin.

[2] Chen W, Zheng R, Baade PD, Zhang S, Zeng H, Bray F, Jemal A, Yu XQ, He J (2016). Cancer statistics in China, 2015. CA Cancer J Clin.

[3] Kapranov P, Cheng J, Dike S, Nix DA, Duttagupta R, Willingham AT, Stadler PF, Hertel J, Hackermüller J, Hofacker IL, Bell I, Cheung E, Drenkow J (2007). RNA maps reveal new RNA classes and a possible function for pervasive transcription. Science.

[4] Wang KC, Yang YW, Liu B, Sanyal A, Corces-Zimmerman R, Chen Y, Lajoie BR, Protacio A, Flynn RA, Gupta RA, Wysocka J, Lei M, Dekker J (2011). A long noncoding RNA maintains active chromatin to coordinate homeotic gene expression. Nature.

[5] Chen LL (2016). Linking Long Noncoding RNA Localization and Function. Trends Biochem Sci.

[6] Loewer S, Cabili MN, Guttman M, Loh YH, Thomas K, Park IH, Garber M, Curran M, Onder T, Agarwal S, Manos PD, Datta S, Lander ES (2010). Large intergenic non-coding RNA-RoR modulates reprogramming of human induced pluripotent stem cells. Nat Genet.

[7] Luo Q, Chen Y (2016). Long noncoding RNAs and Alzheimer's disease. Clin Interv Aging.

[8] Quagliata L, Matter MS, Piscuoglio S, Arabi L, Ruiz C, Procino A, Kovac M, Moretti F, Makowska Z, Boldanova T, Andersen JB, Hämmerle M, Tornillo L (2014). Long noncoding RNA HOTTIP/HOXA13 expression is associated with disease progression and predicts outcome in hepatocellular carcinoma patients. Hepatology.

[9] Ye H, Liu K, Qian K (2016). Overexpression of long noncoding RNA HOTTIP promotes tumor invasion and predicts poor prognosis in gastric cancer. Onco Targets Ther.

[10] Ge Y, Yan X, Jin Y, Yang X, Yu X, Zhou L, Han S, Yuan Q, Yang M (2015). MiRNA-192 and miRNA-204 Directly Suppress lncRNA HOTTIP and Interrupt GLS1-Mediated Glutaminolysis in Hepatocellular Carcinoma. PLoS Genet.

[11] Wang Y, Li Z, Zheng S, Zhou Y, Zhao L, Ye H, Zhao X, Gao W, Fu Z, Zhou Q, Liu Y, Chen R (2015). Expression profile of long non-coding RNAs in pancreatic cancer and their clinical significance as biomarkers. Oncotarget.

[12] Lian YF, Ding J, Zhang Z, Shi Y, Zhu Y, Li J, Peng P, Wang J, Fan Y, De W, Wang K (2016). The long noncoding RNA HOXA transcript at the distal tip promotes colorectal cancer growth partially via silencing of p21 expression. Tumour Biol.

[13] Deng HP, Chen L, Fan T, Zhang B, Xu Y, Geng Q (2015). Long non-coding RNA HOTTIP promotes tumor growth and inhibits cell apoptosis in lung cancer. Cell Mol Biol (Noisy-le-grand).

[14] Zhang H, Zhao L, Wang YX, Xi M, Liu SL, Luo LL (2015). Long non-coding RNA HOTTIP is correlated with progression and prognosis in tongue squamous cell carcinoma. Tumour Biol.

[15] Ren YK, Xiao Y, Wan XB, Zhao YZ, Li J, Li Y, Han GS, Chen XB, Zou QY, Wang GC, Lu CM, Xu YC, Wang YC (2015). Association of long non-coding RNA HOTTIP with progression and prognosis in colorectal cancer. Int J Clin Exp Pathol.

[16] Li F, Cao L, Hang D, Wang F, Wang Q (2015). Long non-coding RNA HOTTIP is up-regulated and associated with poor prognosis in patients with osteosarcoma. Int J Clin Exp Pathol.

[17] Yang Y, Qian J, Xiang Y, Chen Y, Qu J (2017). The prognostic value of long noncoding RNA HOTTIP on clinical outcomes in breast cancer. Oncotarget.

[18] Lian Y, Cai Z, Gong H, Xue S, Wu D, Wang K (2016). HOTTIP: a critical oncogenic long non-coding RNA in human cancers. Mol Biosyst.

[19] Tsang FHC, Au SL, Wei L, Fan DN, Lee JM, Wong CC, Ng IO, Wong CM (2015). Long non-coding RNA HOTTIP is frequently up-regulated in hepatocellular carcinoma and is targeted by tumour suppressive miR-125b. Liver Int.

[20] Li Z, Zhao X, Zhou Y, Liu Y, Zhou Q, Ye H, Wang Y, Zeng J, Song Y, Gao W, Zheng S, Zhuang B, Chen H (2015). The long non-coding RNA HOTTIP promotes progression and gemcitabine resistance by regulating HOXA13 in pancreatic cancer. J Transl Med.

[21] Cheng Y, Jutooru I, Chadalapaka G, Christopher Corton J, Safe S (2015). The long non-coding RNA HOTTIP enhances pancreatic cancer cell proliferation, survival and migration. Oncotarget.

[22] Zhang SR, Yang JK, Xie JK, Zhao LC (2016). Long noncoding RNA HOTTIP contributes to the progression of prostate cancer by regulating HOXA13. Cellular and molecular biology (Noisy-le-Grand, France).

[23] Xiao J, Hu CP, He BX, Chen X, Lu XX, Xie MX, Li W, He SY, You SJ, Chen Q (2016). PTEN expression is a prognostic marker for patients with non-small cell lung cancer: a systematic review and meta-analysis of the literature. Oncotarget.

